# Valor and Skill in the Civil War

**Published:** 1890-06

**Authors:** 


### VALOR AND SKILL IN THE CIVIL WAR.

Two articles in the May *Century*, discuss the relative merits of the blue and the gray in the trials of battle. General Theodore A. Dodge writes to the question, "Was either the better soldier?" and Charles A. Patch asks, "Which was the better army?" In conclusion, General Dodge says that his "list of fifty battles gives twenty victories to the Confederates, an equal number to the Federals, and leaves ten which may fairly be called drawn. In these fifty battles, at the point of fighting contact, the Confederates outnumbered the Federals by an average of about two per cent.

"As regards brilliant assaults upon regular works, the Confederates were never called on to show such devotion as was manifested by the Federals at Fredericksburg, the several assaults at Vicksburg and Port Hudson, Spottsylvania, Cold Harbor and Petersburg. Few trials of fighting qualities, in any war, go beyond some of these.

"As will be seen from the table of forces, after the winter of 1863-64, the Union forces so vastly outnumbered the Confederates, that comparison of the merits of actual fighting becomes more difficult.

We can deduce little from the battles except stanch purpose on the Federal, and brilliant courage, coupled with marvelously able military management, on the Confederate side. But if one will take the pains to tabulate the numbers actually engaged during all but the last months of the crumbling away of the Confederate armies, there appear plainly two facts : first, that the Confederates, by superior management and better position, opposed to the Federals fully equal numbers at the point of fighting contact ; and secondly, that of the combats during the entire struggle the Federals have their full share of the victories.

"It is certain that the statistics of the war rob the wearers of the blue and the gray of the right to boast one at the expense of the other. Neither can claim superiority in actual battle. The case bears enough semblance to Greek meeting Greek, to satisfy the reasonable aspirations of either 'Yank' or 'Johnny.'"

